# Sex-dependent effects of food-restriction on cocaine self-administration and cocaine-seeking in rats

**DOI:** 10.3389/fnbeh.2025.1603564

**Published:** 2025-06-10

**Authors:** Alixandria T. Mascarin, Ava M. Mac, Srinivasu Kallakuri, Mark K. Greenwald, Shane A. Perrine

**Affiliations:** ^1^Department of Psychiatry and Behavioral Neurosciences, School of Medicine, Wayne State University, Detroit, MI, United States; ^2^Department of Pharmacy Practice, Eugene Applebaum College of Pharmacy and Health Sciences, Wayne State University, Detroit, MI, United States

**Keywords:** self-administration, cocaine, biological sex differences, cocaine-seeking, food-restriction

## Abstract

**Introduction:**

Misuse of drugs and natural rewards, such as food, share common neural pathways and comparably influence behavioral consequences. Food-restriction enhances drug-taking and drug-seeking behaviors in animals, but the effect of food-restriction on cocaine self-administration and cocaine-seeking in both sexes has not been well characterized.

**Methods:**

Therefore, the present study investigated differences between food-restricted vs. *ad libitum-*feeding male and female Wistar rats on the acquisition of cocaine self-administration and cue-induced reinstatement of cocaine-seeking.

**Results:**

Food access sex-dependently altered the acquisition of cocaine self-administration such that food-restricted females, but not males, displayed an escalated intake behavior over time. Only food-restricted females differed significantly between active and inactive lever pressing during the reinstatement of cocaine-seeking session.

**Discussion:**

Taken together, these results suggest that food-restriction sex-dependently improves learning of cocaine self-administration that persists to alter cocaine-seeking behavior following abstinence.

## 1 Introduction

Food and drug rewards share common neural substrates (Cardinal and Everitt, 2004; Kelley and Berridge, 2002; Volkow et al., 2008), and manipulation of food access (i.e., food-restriction) is commonly used in animal models of drug self-administration to increase responding (Carr, 2002; Carr, 2020). Food and drug (e.g., cocaine) rewards take effect in the mesolimbic dopamine pathway, and this commonality is reflected in the relationships between feeding and substance use behaviors (Carr, 2020). The impact of food-restriction on opioid taking and seeking behaviors has been well-established. For example, male rats undergoing food-restriction display heightened opioid self-administration relative to *ad libitum-* (i.e., as much or as often as wanted) feeding rodents (Carroll et al., 1979) with reduced body weight being necessary for this effect (Carroll and Meisch, 1980). Chronic food-restriction also increases opioid-seeking behavior in male rat models of relapse (D’Cunha et al., 2013, 2017, 2020). However, research studies examining the impact of food-restriction on cocaine self-administration and the reinstatement of cocaine-seeking are very limited and report mixed results. Bongiovanni & See reported no difference between food-restricted and *ad libitum* feeding male rats on cocaine self-administration, extinction, or reinstatement (Bongiovanni and See, 2008). On the contrary, food-restricted male rats met the criterion for acquisition of cocaine self-administration faster than their food-satiated counterparts (Campbell and Carroll, 2001). Therefore, further exploration is necessary to elucidate the impact of food-restriction on cocaine self-administration and reinstatement.

Importantly, the studies mentioned above only included male animals. However, (biological) sex differences in cocaine self-administration and reinstatement have been reliably observed (Becker and Hu, 2008; Becker and Koob, 2016; Lynch and Carroll, 1999; Lynch and Taylor, 2004; Nicolas et al., 2019; Nicolas et al., 2022; Roth and Carroll, 2004), and other studies have explored sex differences at the intersection of cocaine use and food intake. For example, more female rats choose cocaine over palatable pellets than male conspecifics (Perry et al., 2013), and gonadal sex hormones (e.g., estradiol) may mediate this effect (Kerstetter and Kippin, 2011). Carroll and Campbell found that food-restriction increased heroin self-administration similarly in both sexes; however, ketoconazole (a corticosterone synthesis inhibitor) attenuated this effect in females, but not in males (Carroll et al., 2001). Sedki et al. report an enhancement of opioid-seeking by food-restriction in ovariectomized female rats that is attenuated by estradiol replacement (Sedki et al., 2015). Despite these reports, it remains unclear how food-restriction impacts cocaine self-administration and reinstatement or how sex may modulate that relationship. Therefore, this study compared the behavior of female and male rats subjected either to *ad libitum* or restricted food access on cocaine self-administration and cocaine-seeking following a period of forced abstinence. We hypothesized that food-restriction would increase self-administration and cocaine-seeking in both sexes and that, within each food access condition, females would display greater cocaine self-administration and cocaine-seeking relative to males.

## 2 Materials and methods

### 2.1 Animals

Eighty-six male and female Wistar rats bred at Wayne State University were used for this study. The rats were heterozygous for the Fos-LacZ transgene [Wistar-Tg (Fos-LacZ) 1Ottc], but the transgene was not utilized in this study. The rats were kept on a reversed 12 h light-dark cycle (lights off 6 AM) with otherwise standard environmental and housing conditions. Food-restricted rats (*n* = 22 male, 18 female) were single-housed for the duration of the study, and *ad libitum* feeding rats (*n* = 23 male, 23 female) were pair-housed, except in the infrequent occurrence of cage-mate euthanasia due to post-surgical complications. Water was provided *ad libitum* to all rats except in the testing apparatus. All procedures were approved by the Wayne State University Institutional Animal Care and Use Committee and followed the guidelines in the Guide for the Care and Use of Laboratory Animals (National Research Council, 2011).

### 2.2 Intravenous catheter implantation surgery

Catheter implantation occurred on or around post-natal day 70 as previously described (Eagle et al., 2015; Perrine et al., 2022). Briefly, rats were anesthetized (5% isoflurane in 0.6 L oxygen), and polyurethane catheters (Instech Laboratories) were implanted into the right jugular vein using sterile, aseptic techniques. Catheters were connected to a magnetic vascular access button (Instech Laboratories) implanted posterior to the shoulder blades. Carprofen (5 mg/kg) was dissolved in sterile saline and administered subcutaneously prior to surgery and for the following 3 days. Rats were allowed to recover for 7–10 days before initiation of cocaine self-administration. Throughout the paradigm, catheter systems were flushed weekly with 0.1 mL of 20 USP sterile heparinized saline flush solution (Instech Laboratories). Resistance during flushing would have indicated a loss of patency, but this was not observed in the present study.

### 2.3 Food-restriction

All rats were maintained on *ad libitum* standard rodent chow (LabDiet; 28.9% of calories from protein, 13.6% from fat, and 57.5% from carbohydrates) until 7 days before the paradigm began. The rats were then assigned to either *ad libitum* food-access or food-restriction group. On the first day of food-restriction, food-restricted rats received an initial weaning amount of 20 g (female) or 25 g (male) of regular rodent chow (LabDiet). Subsequently and according to Wayne State University Institutional Animal Care and Use Committee guidelines, they were maintained on 14 g (female) or 20 g (male) of rodent chow for the duration of the study (Bongiovanni and See, 2008; Campbell and Carroll, 2001; Kane et al., 2021; Warren et al., 2019). Food-restricted rats were fed daily, immediately following behavioral sessions.

### 2.4 Drugs

[-]Cocaine HCl was obtained from the National Institute on Drug Abuse (NIDA) Drug Supply Program. Cocaine was dissolved in sterile saline (0.9% NaCl) and infused at a dose of 0.5 mg/kg/infusion on a fixed-ratio 1 schedule of reinforcement with 20-s timeouts between drug-available periods. The cocaine dose was maintained by adjusting the infusion delivery duration on the fixed-rate infusion pump.

### 2.5 Apparatus

Rats underwent cocaine self-administration and cocaine-seeking sessions individually in self-administration chambers (53.34 × 34.93 × 27.31 cm; Med Associates). The chambers were housed in isolation cubicles and equipped with two levers (active and inactive), lever-associated lights, and a red house light. To allow free movement around the chamber, rats were connected to a magnetic tether and swivel system, which included a single-channel vascular access button (Instech Laboratories) surgically implanted posterior to the rats’ shoulder blades. The chambers were equipped with a fixed-rate infusion delivery pump that was connected via a polyurethane tube to the tether system (Med Associates).

### 2.6 Cocaine self-administration and cocaine-seeking sessions

**FIGURE 1:**
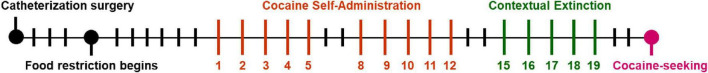
Timeline of experimental procedures. Vertical orange and green lines represent days where self-administration and contextual extinction sessions occurred, respectively. Black lines represent days of no behavioral testing.

**FIGURE 2:**
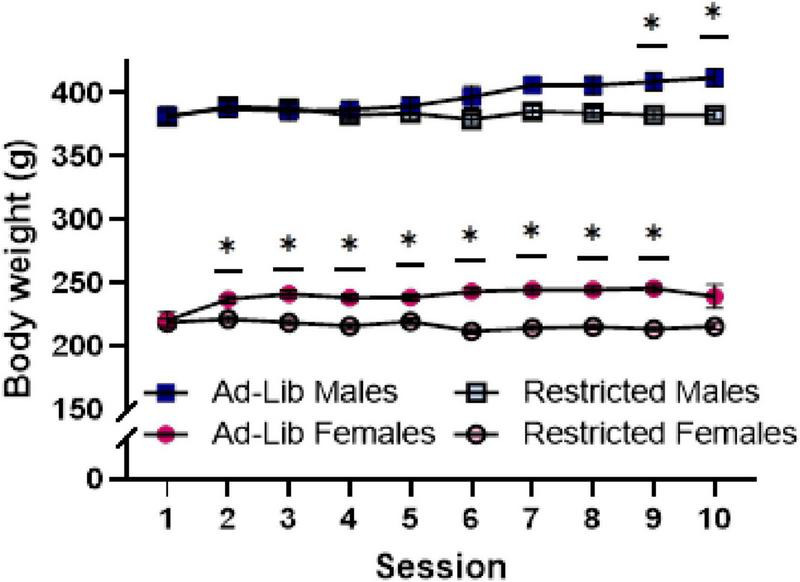
Food-restriction reduces body weight in both male and female rats. Body weight (g), taken immediately before self-administration sessions. Error bars represent the standard error of the mean and are smaller than the symbol where not visible. Ad-Lib, *ad libitum* food access (filled symbols). Restricted, restricted food access (open symbols). Lines indicate comparison within sex and between food access groups (*p < 0.05).

**FIGURE 3:**
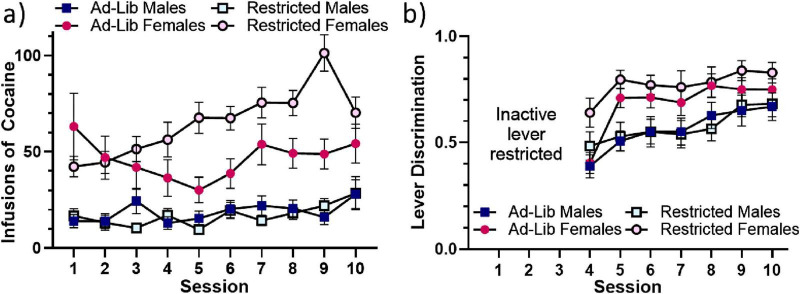
Food-restriction sex-dependently impacts acquisition of cocaine self-administration. **(a)** Mean self-administered infusions of cocaine across the 10 3-h sessions. **(b)** Mean lever discrimination (active lever presses/total lever presses) across sessions 4-10. The inactive lever was restricted for sessions 1-3. Error bars represent standard error of the mean. Ad-Lib, *ad libitum* food access. Restricted, restricted food access.

**FIGURE 4:**
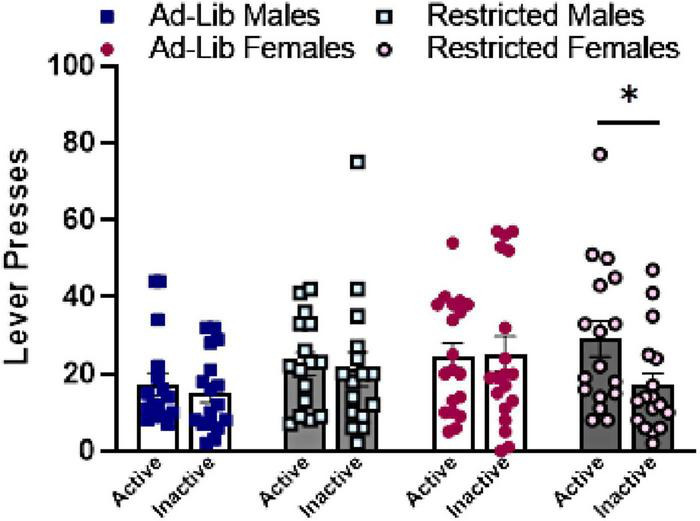
Food-restricted females differ significantly on active and inactive lever pressing during reinstatement of cocaine-seeking. Active and inactive lever presses during the 30-min cocaine-seeking session. Error bars represent the standard error of the mean. Ad-Lib, *ad libitum* food access. Restricted, restricted food access. Individual symbols (squares, circles) represent single rats. Line indicates comparison within group on levers (*p < 0.05).

The experimental paradigm consisted of three phases, as shown in Figure 1: (1) acquisition of cocaine self-administration for 10 sessions over days 1-12, (2) forced abstinence on days 13-21 with 1-h contextual extinction sessions on days 15-19, and (3) a cue-induced cocaine-seeking session on day 22. For each of the 10 self-administration sessions, rats were weighed before the beginning of the session and then allowed to self-administer cocaine for 3 h. Self-administration sessions occurred over 12 days with a 2-day forced abstinence period between sessions 5 and 6. To promote robust acquisition, the inactive lever was retracted during the first three self-administration sessions. However, for the remaining self-administration sessions, both levers were available. During all self-administration sessions, the light associated with the active lever was turned on. Pressing the active lever initiated the infusion pump to deliver cocaine and then induced a 20-s timeout period, during which both the levers were retracted, the lever light was inactivated, and the house light was turned on. Inactive lever presses had no programed consequence but were recorded. The rats were required to meet an acquisition criterion of > 50% active lever pressing during the last 3 self-administration sessions. The 16 rats that failed to reach this criterion (6 food-restricted and 6 *ad libitum* feeding males, 1 food-restricted and 3 *ad libitum* feeding females) are included in the acquisition analyses (Figures 2, 3) but were excluded from the study following acquisition (Figure 4; Supplementary Figure 1). During forced abstinence periods, the rats were confined to their home cage, except during the 1-h extinction sessions on days 15-19, which took place in the self-administration chamber, resulting in a total of 6 non-drug, non-testing days. During extinction sessions, the rats were placed in the chamber but not presented with any cocaine-associated cues or levers. Finally, the cocaine-seeking session (30 min) on day 22 was induced by the presentation of cocaine-associated cues and conducted under the same conditions as the self-administration sessions (3 h), except that active lever presses did not deliver cocaine.

### 2.7 Statistical analyses

Statistical analyses were performed using IBM SPSS version 29 and GraphPad Prism 10 and employed a significance value threshold of *p* < 0.05. To assess body weight differences, a three-way mixed-model ANOVA used sex and food access as independent variables, sessions as the repeated measure, and weight in grams as the dependent variable. Cocaine self-administration was analyzed with a mixed-effects model, using sex and food access as independent variables, session as the repeated measure, and the number of infusions or lever discrimination (number of active lever presses/total lever presses) as the dependent variables. Because the assumption of sphericity was violated in this sample, the Huynh-Feldt correction was utilized for all ANOVAs. Cocaine-seeking was analyzed by three-way ANOVA with sex, food access, and lever (active vs. inactive) as the independent variables and lever presses as the dependent variable. Šídák’s multiple comparisons test was used for *post hoc* analysis of body weight, cocaine self-administration, and cocaine-seeking. A chi-square analysis was used to assess sex bias in meeting the acquisition criterion.

## 3 Results

### 3.1 Effect of food-restriction on body weight

As expected, food-restricted rats weighed less during the self-administration period as indicated by a main effect of food access [*F*(1, 82) = 14.897, *p* < 0.001] and a two-way interaction of food access and session [*F*(9, 594) = 12.78, *p* < 0.0001]. Weight increased across sessions for *ad libitum* but not food-restricted rats, which is supported by a two-way interaction of session and food access [*F*(3.579, 293.502) = 18.379, *p* < 0.001]. Body weight under these conditions was also impacted by sex, as evidenced by a main effect of sex [*F*(1,82) = 1263.768, *p* < 0.001] (Figure 2). Food restriction impacted males and females differently over time, as evidenced by a three-way interaction of session, food access, and sex [*F*(9, 738) = 3.788, *p* = 0.0001]. *Post hoc* comparisons revealed that females’ body weights significantly differed according to food access as early as session 2 (*p* = 0.0055). However, the weight of males differed by food access only in sessions 9 and 10 (*p* = 0.0342).

### 3.2 Cocaine self-administration

A mixed-effects analysis of self-administered infusions of cocaine shown in Figure 3a revealed main effects of sex [female > male: *F*(1, 81) = 58.036, *p* < 0.001] and session [increasing over time: *F*(5.751, 465.857) = 5.197, *p* < 0.001]; two-way interactions of session and sex [*F*(5.751, 465.857) = 2.304, *p* = 0.036], session and food access [*F*(5.751, 465.857) = 3.693, *p* = 0.002], and food and sex [*F*(1, 81) = 4.568, *p* = 0.036]; and a three-way interaction of session, food access, and sex [greater effect of food access across sessions in females: *F*(5.751, 465.857) = 3.679, *p* = 0.002]. Analysis of lever discrimination (% active lever responding) shown in Figure 3b indicated main effects of sex [females > males: *F*(1, 82) = 14.493, *p* < 0.001] and session [increasing over time: *F*(5.338, 473.705) = 13.366, *p* < 0.001]. Finally, females were more likely to meet the acquisition criterion than males, as indicated by a chi-square analysis [*X*^2^(1, 86) = 4.051, *p* = 0.044] (data not shown graphically).

### 3.3 Cocaine-seeking

During the cocaine-seeking session (Figure 4), there was a main effect of lever (active > inactive) [*F*(1,65) = 4.020, *p* = 0.0491]. Only food-restricted females differed significantly between active and inactive lever presses during the cocaine-seeking session [*t*(65) = 3.137, *p* = 0.0303], but sex and food-restriction otherwise did not affect cocaine-seeking.

## 4 Discussion

The present study assessed the impact of standard-chow food-restriction on the acquisition of cocaine self-administration, and cue-induced reinstatement of cocaine-seeking following a drug-free period, in female and male rats. Our findings reveal that food-restriction enhanced cocaine self-administration over time in females, but not in males. Reduced body weight is necessary for food-restriction’s enhancement of opioid self-administration (Carroll and Meisch, 1980). This relationship has not yet been assessed for cocaine self-administration, but our data suggest that the sex-dependent impact of food-restriction on body weight in our sample may have promoted its sex-dependent effect on self-administration. Our data do not show a decrease in body weight of food-restricted animals over time – rather an increase in body weight of *ad libitum* feeding rats, leading to group differences – but body weight may still impact cocaine use behaviors. Perhaps a more stringent food-restriction, such as the 15 g daily allotment for males employed by D’Cunha et al. (2013), may have produced more robust effects on body weight and/or cocaine-related behaviors.

Notably, sex differences in cocaine self-administration and reinstatement can be dose-dependent (Becker and Chartoff, 2019; Datta et al., 2018; Fuchs et al., 2005), and we therefore chose a moderate dose of 0.5 mg/kg/infusion that reportedly minimizes sex differences (Fuchs et al., 2005). In female rats, food-restriction decreases gonadal hormone (e.g., estradiol, follicular-stimulating hormone) levels (Ahmed et al., 2012). Leptin and ghrelin levels are also impacted by food restriction, though findings are mixed (Ahmed et al., 2012; D’Cunha et al., 2020). Thus, reproductive and orexigenic/metabolic hormone signaling may explain our sex-dependent results. Conversely, *ad libitum* food intake may have reduced vulnerability to cocaine use behaviors in females. To this point, blockade of appetite suppressant leptin signaling in the NAc enhances cocaine-conditioned reward (Shen et al., 2016).

We identified a main effect of lever in the cocaine-seeking session, suggesting that the rats in this study successfully reinstated cocaine-seeking. However, this effect was driven by food-restricted females, which was the only group that differed significantly in the *post hoc* analysis of active and inactive lever presses. This result suggests that food-restriction in females, but not males, may promote stronger learning that persists to the cocaine-seeking session. Indeed, food-restriction increases memory persistence in spatial learning tasks (Talhati et al., 2014) and increases reinstatement of reward-related learning (Carr, 2011; Zheng et al., 2012), though the intersection of food access and biological sex remains understudied.

While our study identified a sex-dependent impact of food-restriction on acquisition of cocaine-self-administration, we found little evidence to support an impact on reinstatement of cocaine-seeking. In contrast, food-restriction reliably potentiates opioid self-administration and opioid-seeking (Carroll and Meisch, 1980; Carroll et al., 1979; Carroll et al., 2001; D’Cunha et al., 2013, 2017, 2020). A number of differences between opioids and psychostimulants may contribute to these discordant findings and are reviewed in Badiani et al. (2011). As methodological considerations (e.g., dose, acquisition period, extinction vs. abstinence, etc.) can impact findings, additional research exploring the impact of food-restriction on models of cocaine use in both sexes is warranted to determine whether food-restriction differentially affects behavioral responses to these two drug classes.

Despite best efforts to the contrary, this study has some limitations. For example, acquisition criterion was relatively liberal, despite being more conservative than studies that employ lower or no discrimination threshold in their acquisition criteria (Lacy et al., 2018; Mojica et al., 2014). We chose this criterion to avoid artificially excluding differences that might naturally arise between groups while only including rats that performed better than chance. Additionally, *ad libitum* rats were pair-housed when possible, and food-restricted rats were single-housed by necessity to control food dosing. Consistent housing among groups would have been ideal, but Westenbroek and colleagues found that, relative to pair-housing, single housing does not impact cocaine self-administration on a fixed-ratio 1 schedule in males or females (Westenbroek et al., 2013). Additionally, single-housed rats in this study were given an extra piece of enrichment to minimize housing stress (Simpson and Kelly, 2011). Although we do not think that this limitation substantially impacted our results, male rats socially isolated through adolescence (post-natal days 22-51) showed greater amphetamine-seeking in adulthood (Garcia and Cain, 2021). While single-housing alone does not impact acquisition of cocaine self-administration, it is possible that it may have an effect in combination with food-restriction, and future studies could systematically address this question. Finally, we did not measure gonadal hormones in this study, despite evidence suggesting that gonadal hormones influence cocaine self-administration (Jackson et al., 2006) and are impacted by food-restriction (Ahmed et al., 2012).

In summary, we observed that food-restriction impacted the acquisition of cocaine self-administration in females, but not males, and that it had little impact on cocaine-seeking. We speculate that this impact derives from early improvements in learning relevant to the operant task and that this learning may persist to improve lever discrimination during cocaine-seeking for females, but not males. Though these results highlight the importance of assessing the effects of experimental conditions such as food access (and potentially the energy density of available food) in both sexes, the molecular mechanisms that impact this sex-dependent influence of food-restriction are poorly understood and should be further explored.

## Data Availability

The raw data supporting the conclusions of this article will be made available by the authors, without undue reservation.

## Supplementary material

Supplementary Figure 1 **(a)** active the 10 3-h sessions and **(b)** inactive lever presses across sessions 4-10. The inactive lever was restricted for sessions 1-3. Error bars represent standard error of the mean. Ad-Lib: *ad libitum* food access. Restricted: restricted food access.
