# Supplementary figures and images for: Sex-dependent effects of food-restriction on cocaine self-administration and cocaine-seeking in rats
Source: Front Behav Neurosci. 2025 Jun 10;19:1603564. doi: 10.3389/fnbeh.2025.1603564 (PMC12185388; doi:10.3389/fnbeh.2025.1603564)

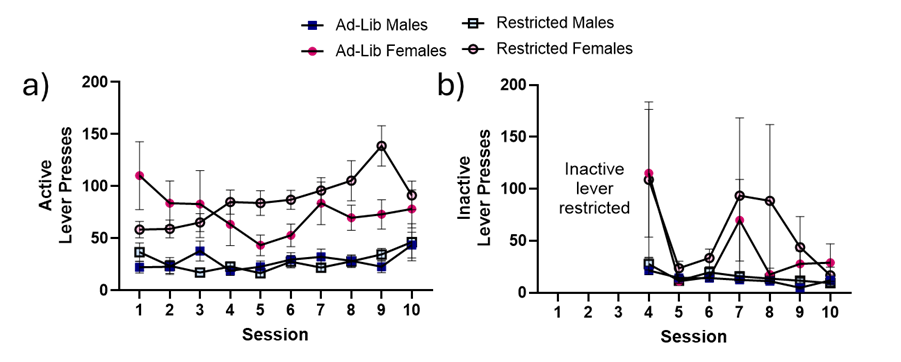

Supplement: Supplementary Figure 1 — (a) active the 10 3-h sessions and (b) inactive lever presses across sessions 4-10. The inactive lever was restricted for sessions 1-3. Error bars represent standard error of the mean. Ad-Lib: ad libitum food access. Restricted: restricted food access. [file Image_1.png]
